# Effect of Plasma Excitation Mode on Rutile TiO_2_ MIM Capacitors Grown on RuO_2_ Seed Electrodes

**DOI:** 10.3390/nano16181151

**Published:** 2026-09-14

**Authors:** Yongwoon Jang, Byungwook Kim, Hyeonwu Nam, Minkyun Kang, Changyun Hong, Changbun Yoon

**Affiliations:** Department of Advanced Materials Engineering, Tech University of Korea, Siheung 15073, Gyeonggi, Republic of Korea; jyw5414@tukorea.ac.kr (Y.J.); quddnr000@tukorea.ac.kr (B.K.); hyeonwu0604@tukorea.ac.kr (H.N.); dakldsa@tukorea.ac.kr (M.K.); jake3121@tukorea.ac.kr (C.H.)

**Keywords:** remote plasma ALD, TiO_2_ high-k dielectric, MIM capacitor, rutile phase, RuO_2_ electrode, DRAM

## Abstract

Maintaining low leakage current with high capacitance density in metal–insulator–metal (MIM) capacitors is essential for next-generation dynamic random-access memory (DRAM) scaling. Rutile TiO_2_ is a promising high-k dielectric; however, its narrow bandgap causes high leakage, while defect-free stabilization in ultrathin films remains challenging. RuO_2_/TiO_2_/Ru MIM capacitors were fabricated using a reactive direct current (DC)-sputtered RuO_2_ bottom electrode, followed by TiO_2_ growth by direct plasma atomic layer deposition (DP-ALD) or remote plasma atomic layer deposition (RP-ALD) and rapid thermal annealing in O_2_ to reduce defects. Rutile TiO_2_ was deposited directly on highly crystalline RuO_2_ under both plasma modes, suggesting that RuO_2_ crystallinity governs TiO_2_ phase evolution. The RP-ALD film replicated the RuO_2_ grain morphology, yielding higher roughness than that of the DP-ALD film. Moreover, the RP-ALD film exhibited lower oxygen-vacancy density and improved stoichiometric stability. The RP-ALD-fabricated capacitors exhibited a higher dielectric constant and lower leakage current density at 0.8 V than the DP-ALD-fabricated capacitors (~100 and ~1.76 × 10^−6^ A/cm^2^ vs. ~97 and ~1.41 × 10^−4^ A/cm^2^, respectively). Ion bombardment during DP-ALD likely promoted oxygen-vacancy-related defect formation, whereas RP-ALD mitigated such damage, improving leakage characteristics. This work highlights the potential of RP-ALD-based RuO_2_/TiO_2_/Ru MIM capacitors for next-generation DRAM.

## 1. Introduction

Dynamic random-access memory (DRAM) is widely used as the main memory in modern computing systems [1,2]. As the demand for higher memory bandwidth and storage density increases, DRAM technology continues to be miniaturized to accommodate more memory cells within a given chip area [3]. Each DRAM cell consists of a transistor and capacitor, with data represented by the presence or absence of charge stored in the capacitor. Because stored charge is lost through leakage current, DRAM cells require periodic refresh operations; hence, suppressing leakage while maintaining sufficient capacitance becomes essential for stable device operation [4]. Accordingly, as device dimensions shrink, new materials and advanced deposition techniques capable of maintaining high capacitance are being actively developed to improve chip yield and extend refresh intervals [5].

As DRAM scales to sub-10 nm technology nodes, achieving high capacitance density within an increasingly limited cell footprint becomes progressively challenging [6]. Because the capacitance of a metal–insulator–metal (MIM) capacitor is determined by the dielectric constant and thickness of the dielectric layer, adopting high-k dielectric materials is a key strategy for maintaining adequate charge storage capacity [7,8]. Currently, ZrO_2_/Al_2_O_3_/ZrO_2_ (ZAZ) composite thin films are widely used as the standard dielectric for DRAM capacitors. However, continued miniaturization has brought ZAZ-based structures close to a physical limit, beyond which further thickness scaling is difficult and ultimately reduces charge storage capacity [9,10]. To overcome this limitation, alternative high-k materials such as HfO_2_, ZrO_2_, Al-doped TiO_2_, SrTiO_3_, and TiO_2_ have been extensively investigated as potential replacements, each offering distinct advantages in relation to the dielectric constant, bandgap, and process compatibility [11,12,13].

Among these candidates, rutile TiO_2_ has attracted particular attention as a promising high-k dielectric for next-generation DRAM capacitors, owing to its high dielectric constant (>80–100) [14,15]. However, two fundamental issues hinder its practical implementation. First, rutile TiO_2_ has a relatively narrow bandgap (~3.0 eV), which intrinsically increases leakage current and can degrade device reliability [14,16]. Second, stabilizing the high-k rutile phase in ultrathin films is challenging. TiO_2_ preferentially nucleates as the low-k anatase phase under typical low-temperature deposition conditions [17,18], and the anatase-to-rutile phase transition generally requires high temperatures that may be incompatible with back-end-of-line constraints. Addressing these issues requires careful optimization of both the electrode material and deposition process [19,20].

Selecting the bottom electrode is critical for the phase evolution of TiO_2_. RuO_2_, which adopts the rutile crystal structure and has lattice parameters similar to those of rutile TiO_2_, is an effective crystallographic seed layer that promotes rutile nucleation and growth [15,21]. The crystallinity of the RuO_2_ electrode strongly depends on deposition conditions, including oxygen partial pressure and substrate temperature. This crystallinity directly determines the degree of rutile TiO_2_ stabilization achievable in the overlying dielectric layer [22,23,24]. Specifically, well-crystallized RuO_2_ can provide an epitaxial-like template that lowers the activation energy required for rutile TiO_2_ nucleation, enabling rutile stabilization even at reduced deposition temperatures [18,25]. Leveraging this structural compatibility enables the formation of high-k rutile TiO_2_ in ultrathin films without relying solely on high-temperature annealing.

For the deposition of ultrathin high-k dielectric films with precise thickness control, atomic layer deposition (ALD) has become a standard process in DRAM manufacturing [26,27]. ALD enables monolayer-level thickness control and uniform deposition through self-limiting surface reactions. Plasma-enhanced atomic layer deposition (PE-ALD) offers additional advantages over thermal ALD, including lower process temperature, higher film density, and improved oxidation efficiency, making it attractive for high-k dielectric deposition [28,29]. PE-ALD is further classified into direct plasma ALD (DP-ALD) and remote plasma ALD (RP-ALD). In DP-ALD, plasma is generated directly within the reaction chamber, providing high reactivity. However, the substrate is exposed to high-energy ion bombardment, which induces surface defects and increases oxygen-deficient defects, potentially degrading film quality [30,31]. In contrast, RP-ALD spatially separates the plasma-generation region from the deposition region, delivering primarily reactive radicals to the substrate and thereby substantially reducing ion-induced damage [32,33,34].

This distinction is particularly important for defect-sensitive high-k dielectrics such as TiO_2_. Accordingly, the plasma deposition process is a key variable that governs not only film composition and defect density but also the electrical performance of the MIM capacitor [35,36].

In this study, RuO_2_/TiO_2_/Ru MIM capacitors were fabricated and the structural and electrical properties of TiO_2_ thin films deposited by DP-ALD and RP-ALD were systematically compared. The RuO_2_ bottom electrode was deposited by reactive direct current (DC) sputtering at various Ar/O_2_ flow ratios and under different substrate temperature conditions to tune electrode crystallinity. TiO_2_ dielectric layers were subsequently deposited using the direct and remote plasma modes. The structural, compositional, and surface properties of the resulting films, together with the electrical characteristics of the MIM capacitors, were comprehensively analyzed. To date, quantitative comparisons of the effects of the plasma excitation mode (direct versus remote) on defect density and leakage characteristics of rutile TiO_2_ grown on a RuO_2_ seed layer within an identical device structure remain limited. To bridge the knowledge gap, this study examines the influence of the plasma excitation mode on film quality and electrical reliability and seeks to contribute to the development of high-k rutile TiO_2_ for next-generation DRAM capacitors [37,38].

## 2. Materials and Methods

### 2.1. Fabrication of MIM Capacitor Devices

**Figure 1:**
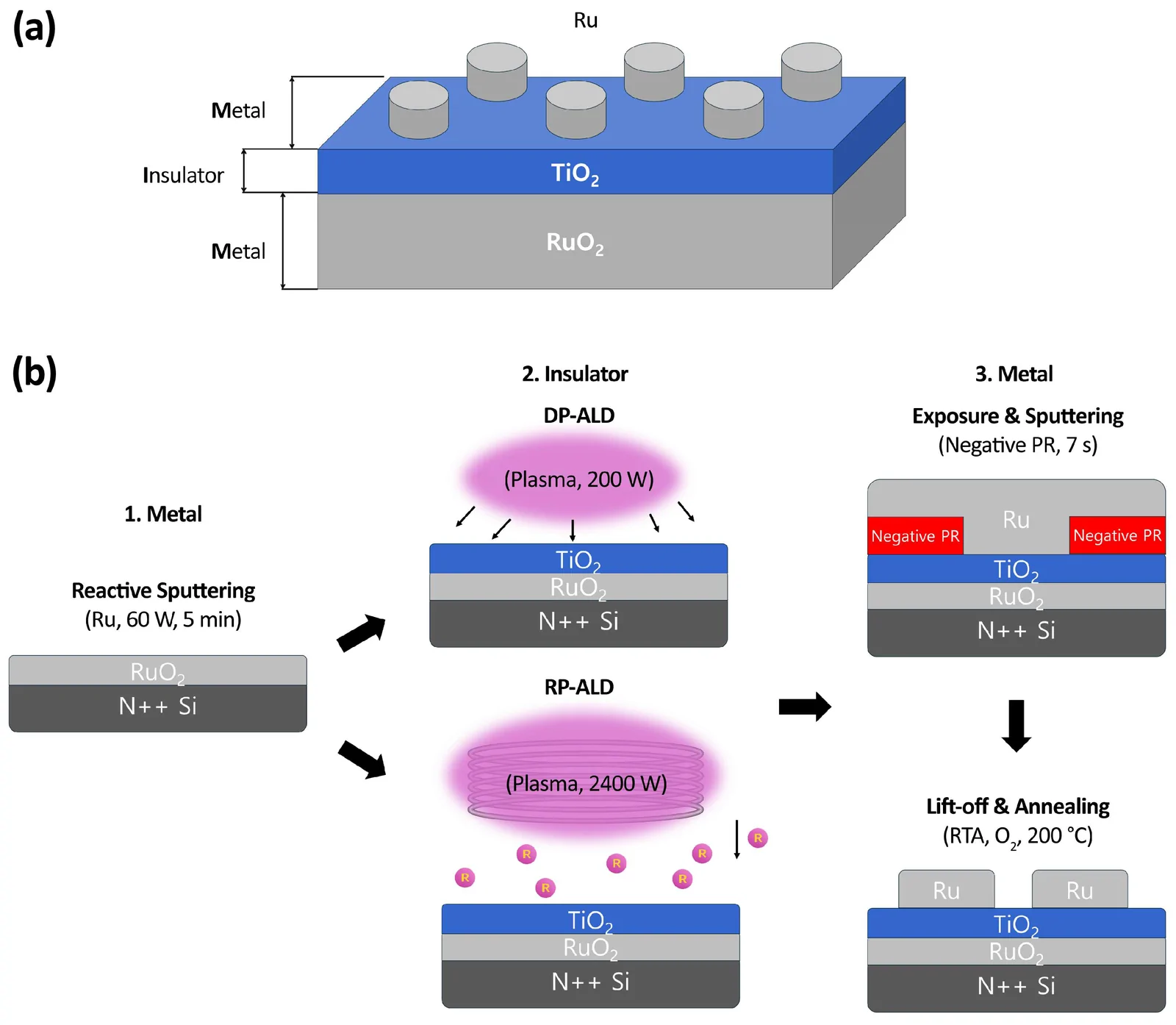
Structure and fabrication process of the TiO_2_-based metal–insulator–metal capacitor. (**a**) MIM capacitor structure. (**b**) Process flow for TiO_2_ dielectric layer deposition using direct plasma atomic layer deposition (DP-ALD) and remote plasma atomic layer deposition (RP-ALD).

Figure 1a shows the structure of the MIM capacitor, comprising a RuO_2_ bottom electrode (metal), a TiO_2_ dielectric layer (insulator), and a circularly patterned Ru top electrode (metal) stacked sequentially. The RuO_2_ bottom electrode was selected for its high conductivity and stability under oxidizing conditions [23]. TiO_2_, which exhibits high-k characteristics, was deposited on top as the dielectric layer to enhance the device capacitance [18,21]. Ru was used as the top electrode and patterned into multiple circular dots (Figure 1a) using a lift-off process. Figure 1b shows the MIM capacitor fabrication process, which comprises three steps: bottom electrode deposition, dielectric layer deposition, and top electrode patterning and deposition.

The RuO_2_ bottom electrode was deposited on N^++^ Si substrates by reactive sputtering using a Ru target (99.99% purity, VTM, Incheon, Republic of Korea) and a DC sputter system (RVS-S100, RAYVAC, Gimpo, Republic of Korea). To identify the optimal process conditions, the substrate temperature (room temperature (RT)–300 °C), Ar flow rate (50–100 sccm), O_2_ flow rate (0–50 sccm), and Ar:O_2_ flow ratio (10:0–7:3) were varied, after which deposition was performed for 5 min at 300 °C and an Ar:O_2_ flow ratio of 7:3 with 60 W sputtering power. These conditions yielded a RuO_2_ bottom electrode with a thickness of approximately 32 nm.

The TiO_2_ dielectric layer was deposited using a PE-ALD system (iOV DX2, ISAC, Daejeon, Republic of Korea) with titanium tetraisopropoxide (TTIP, iChems, Hwaseong, Republic of Korea) as the precursor and O_2_ as the oxygen source. The process pressure was maintained at approximately 1.5 Torr during deposition. To confirm the self-limiting behavior of the ALD process, the source pulse time (0.2–2 s) and purge time (1–10 s) were varied to determine appropriate process conditions. To compare TiO_2_ films deposited by DP-ALD and RP-ALD, films were prepared using both methods. In the DP-ALD process, plasma was generated directly within the PE-ALD chamber with a plasma power of 200 W, and the reactive gas (O_2_) was introduced near the substrate. In the RP-ALD process, a remote plasma source (En2ra-RPS, EN2CORE Technology, Daejeon, Republic of Korea) was used to separate the plasma-generation region from the reaction chamber, such that only radicals activated in the remote plasma region were introduced into the chamber. To ensure sufficient radical density, a remote plasma of approximately 2400 W was applied, and Ar was used as the carrier gas to transport the radicals into the reaction chamber. The TiO_2_ thin films were deposited at 300 °C with target thicknesses of 6, 9, 12, and 15 nm. Film thickness was measured using a spectroscopic ellipsometer (Elli56, Ellipso Technology, Suwon, Republic of Korea).

For the top electrode, a negative photoresist was coated and patterned by UV exposure for 7 s, followed by development using an exposure system (MDA-600LJ, Midas, Daejeon, Republic of Korea). Ru was then deposited by sputtering at 60 W for 5 min, and circular top electrodes with a diameter of 200 μm and a thickness of 40 nm were formed by lift-off. Subsequently, rapid thermal annealing (PT-21PS066, PyroTech, Namyangju, Republic of Korea) was performed at 200 °C in an O_2_ atmosphere to reduce defects in the TiO_2_ dielectric layer and improve its electrical characteristics [39].

### 2.2. Structural and Electrical Characterization

The structural and electrical characteristics of the fabricated MIM capacitors were examined using various techniques. The morphology and cross-sectional structure of the RuO_2_ films were analyzed by scanning electron microscopy (SEM; Apreo 2S, Thermo Fisher Scientific, Waltham, MA, USA) at an accelerating voltage of 2.00 kV. The stacked structure and interfacial characteristics of the MIM capacitors were studied by transmission electron microscopy and energy-dispersive X-ray spectroscopy (TEM/EDS; Talos F200X, Thermo Fisher Scientific, Waltham, MA, USA) at an accelerating voltage of 200 kV. Cross-sectional specimens were prepared using a focused ion beam (SciOS 2, Thermo Fisher Scientific, Waltham, MA, USA).

Film crystallinity and phase were analyzed using X-ray diffraction (XRD; SmartLab, Rigaku, Tokyo, Japan). The RuO_2_/TiO_2_ films were characterized by grazing-incidence X-ray diffraction (GIXRD) using a Cu Kα source (λ = 1.5406 Å). The incidence angle was fixed at 0.3–0.5°, and scans were collected over a 2θ range of 20–60° with a step size of 0.02° and a scan speed of 2°/min. Surface roughness was compared for samples fabricated by DP-ALD and RP-ALD using atomic force microscopy (AFM; XE100, PSIA, Suwon, Republic of Korea).

The composition and defect states of TiO_2_ films deposited by DP- and RP-ALD were analyzed by X-ray photoelectron spectroscopy (XPS; NEXSA, Thermo Fisher Scientific, Waltham, MA, USA). High-resolution XPS spectra were acquired to assess the Ti oxidation state and chemical bonding environment. Measurements were performed using a monochromatic Al Kα source (hν = 1486.6 eV), with an energy resolution of 1.00 eV and a pass energy of 200.0 eV. The X-ray spot size was 400 µm. Surface analysis was conducted after Ar ion etching (500 eV, 40 s) to remove surface contamination. Background subtraction (Shirley method) and spectral fitting (asymmetric Gaussian–Lorentzian) were performed using CasaXPS (version 2.3.19), and the binding energies were calibrated to the C 1s peak (284.8 eV).

Electrical characteristics were measured at RT in the dark by connecting a microprobe station (APX-6B, WIT Co., Suwon, Republic of Korea) to a semiconductor characterization system (4200A-SCS, Keithley, Cleveland, OH, USA). The electrode area was 3.1416 × 10^−4^ cm^2^. Capacitance–voltage (C–V) measurements were performed at 1 MHz, and leakage current density–voltage (J–V) measurements were collected from −1 to 1 V in 0.05 V intervals.

## 3. Results and Discussion

### 3.1. Characteristics of RuO_2_ and TiO_2_ Films

**Figure 2:**
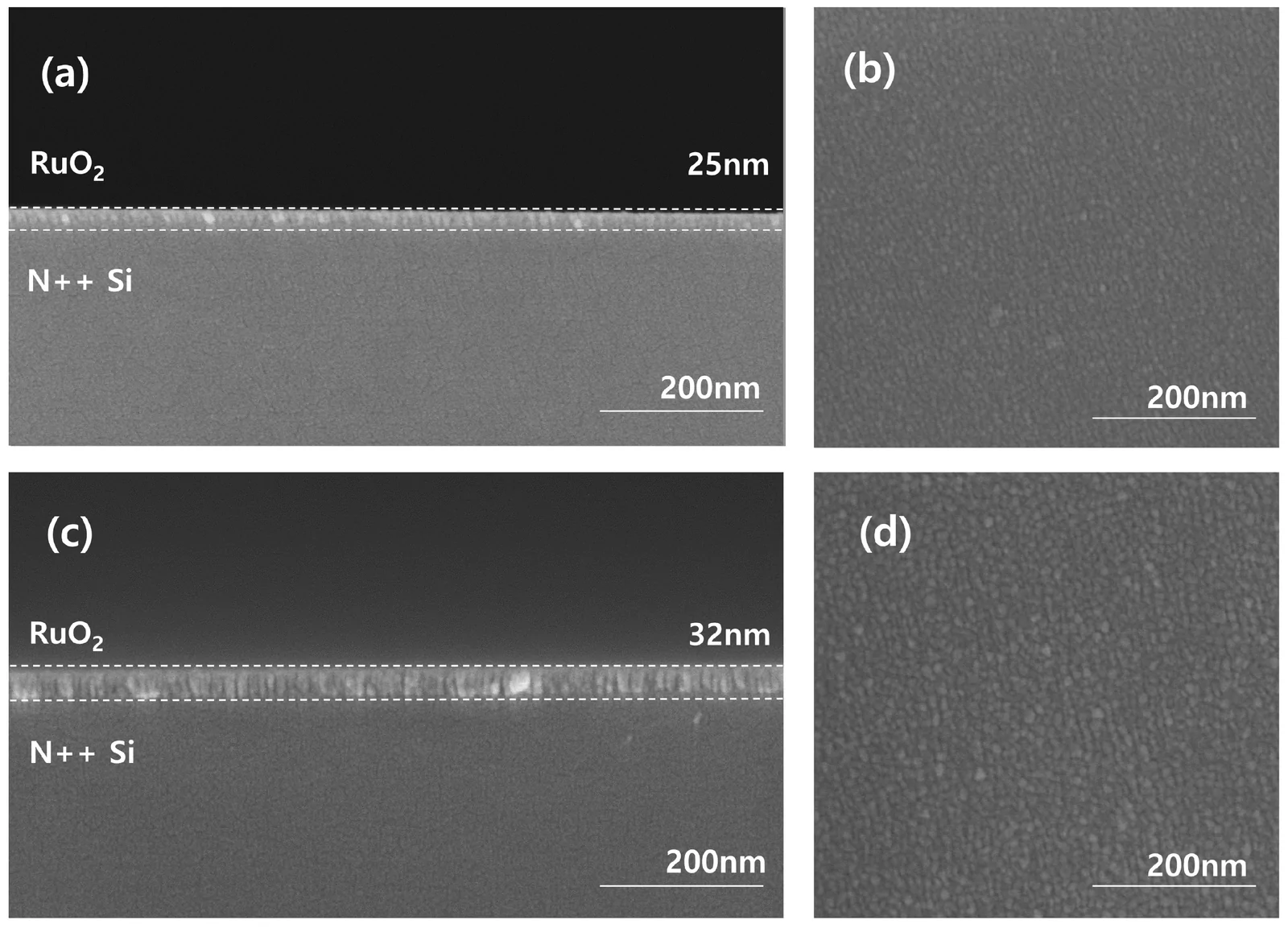
Scanning electron microscopy (SEM) images of the RuO_2_ films deposited at different temperatures. (**a**,**b**) Cross-sectional and surface images, respectively, of the film deposited at room temperature (RT). (**c**,**d**) Cross-sectional and surface images, respectively, of the film deposited at 300 °C.

Figure 2 shows the cross-sectional and surface SEM images of the RuO_2_ films deposited on the N^++^ Si substrates by reactive DC sputtering at a fixed Ar:O_2_ flow ratio of 7:3. To evaluate the effect of the deposition temperature on the film microstructure, deposition was performed under two conditions—RT and 300 °C—and the corresponding cross-sectional and surface morphologies were examined and compared. For a direct comparison, the specimens were deposited for the same duration (5 min) under both conditions.

Figure 2a shows a cross-sectional image of the RuO_2_ film deposited at RT; the film thickness was approximately 25 nm. The film exhibits a dense internal structure, with no observable voids or pores, and the film/substrate interface is distinct and smooth. In the corresponding surface image (Figure 2b), no clear grain boundaries are observed, and the surface exhibits an overall smooth and uniform morphology.

In contrast, Figure 2c shows a cross-sectional image of the RuO_2_ film deposited at 300 °C; the film thickness is approximately 32 nm, which is greater than that obtained at RT. The cross-sectional image indicates the onset of fine columnar features along the vertical direction. The film remains well adhered, with no evidence of delamination from the substrate even at 300 °C. In the corresponding surface image (Figure 2d), unlike those in the film deposited at RT, fine grains with a relatively uniform size distribution are observed in the film deposited at 300 °C, and grain boundaries are clearly resolved. These features indicate more active surface grain growth at higher temperatures, consistent with improved RuO_2_ crystallinity. In addition, the surface roughness of the film deposited at 300 °C increases relative to that of the RT film, supporting a transition toward a crystalline microstructure via nucleation and growth [40].

**Figure 3:**
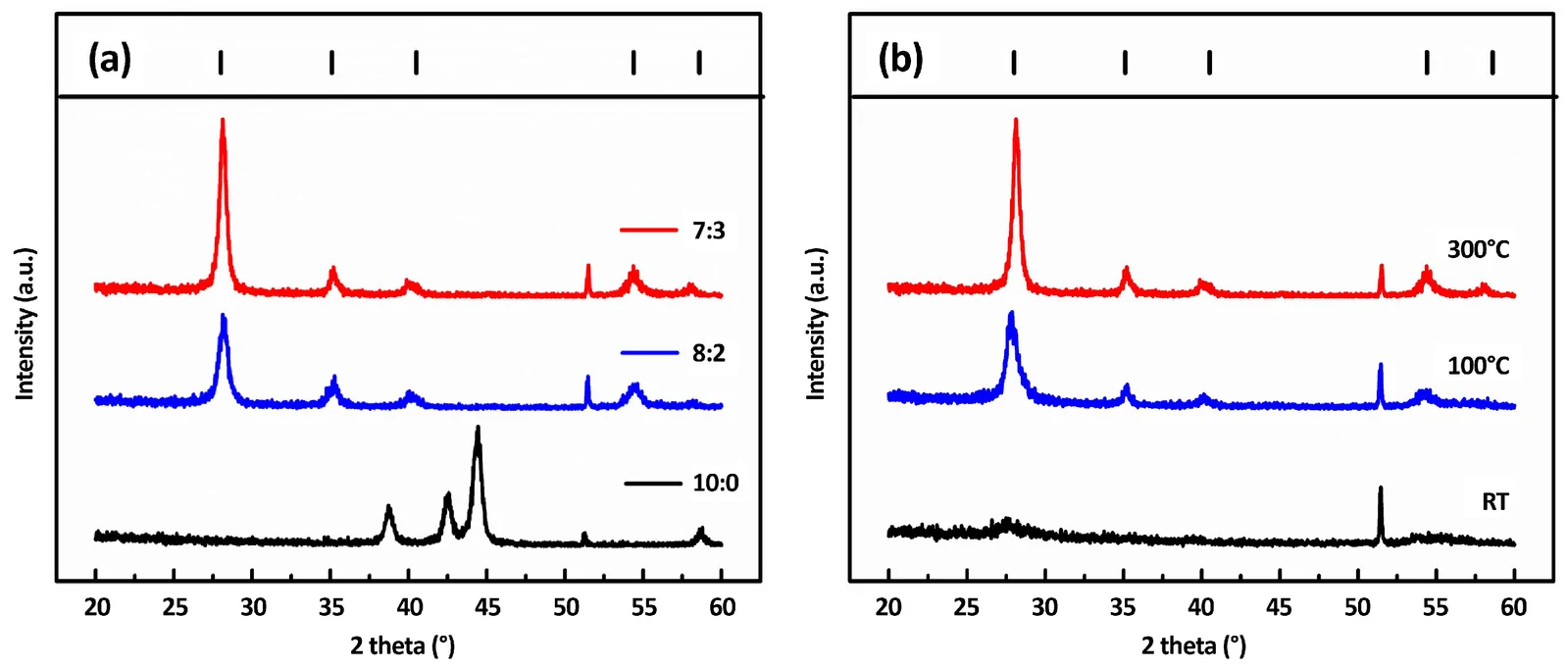
Grazing-incidence X-ray diffraction (GIXRD) patterns for the RuO_2_ films under reactive sputtering conditions. Dependence on the (**a**) Ar/O_2_ flow ratio at 300 °C (10:0, 8:2, 7:3) and (**b**) deposition temperature at Ar/O_2_ = 7:3 (RT, 100 °C, and 300 °C).

Figure 3 shows the GIXRD results used to examine the crystal structure of RuO_2_ films as a function of reactive sputtering conditions. Changes associated with the Ar/O_2_ ratio and deposition temperature are compared.

Figure 3a presents the GIXRD patterns obtained at different Ar/O_2_ ratios at 300 °C. Under the 10:0 condition (pure Ar), no RuO_2_ peaks are observed; instead, several sharp peaks appear near 38°, 42°, 44°, and 58°, corresponding to hexagonal close-packed (hcp) Ru metal, indicating the deposition of a metallic Ru film without oxidation. Under the 8:2 condition, the Ru metal peaks disappear while broad, weak peaks associated with rutile RuO_2_—(110), (101), (200), (211), and (220)—emerge near 28°, 35°, 40°, 54°, and 58°. With a further increase in oxygen partial pressure (7:3), the RuO_2_ peaks at these positions increase markedly in intensity with reduced full width at half maximum (FWHM), indicating the formation of a single-phase RuO_2_ film with improved crystallinity.

This phase evolution is likely attributable to increasing oxygen partial pressure in the deposition ambient. At an Ar/O_2_ ratio of 10:0, oxygen is not available for reaction, and Ru is deposited in a metallic state. At 8:2, partial oxidation occurs, and the rutile RuO_2_ phase begins to form; however, limited crystallinity is reflected in the low-intensity, broadened peaks. At 7:3, the film becomes sufficiently oxidized, and the RuO_2_ phase develops further; the increased (110) peak intensity and reduced full width at half maximum indicate concurrent grain growth and improved crystallinity [24].

Figure 3b shows the GIXRD patterns obtained at different deposition temperatures under the Ar/O_2_ = 7:3 condition. At RT, the RuO_2_ (110) peak near 28° is barely detectable, and the overall pattern is close to a flat baseline without distinct crystalline peaks, indicating that the film is nearly amorphous. When the deposition temperature is increased to 100 °C, the RuO_2_ (110) peak emerges near 28°, and additional RuO_2_-related peaks appear near 35°, 40°, and 54–58°, indicating the onset of crystallization. At 300 °C, the (110) peak exhibits the highest intensity and narrowest linewidth, and the other RuO_2_ peaks are also most clearly resolved. These results indicate that the increased thermal energy enhances atomic diffusion on the growing surface, promoting preferred orientation along the (110) plane and, based on the Scherrer equation, an increase in the grain size. Overall, as the deposition temperature increased from RT to 300 °C, the RuO_2_ film evolved from a nearly amorphous state to a crystalline structure with a (110) preferred orientation, with the highest crystallinity obtained at 300 °C.

**Figure 4:**
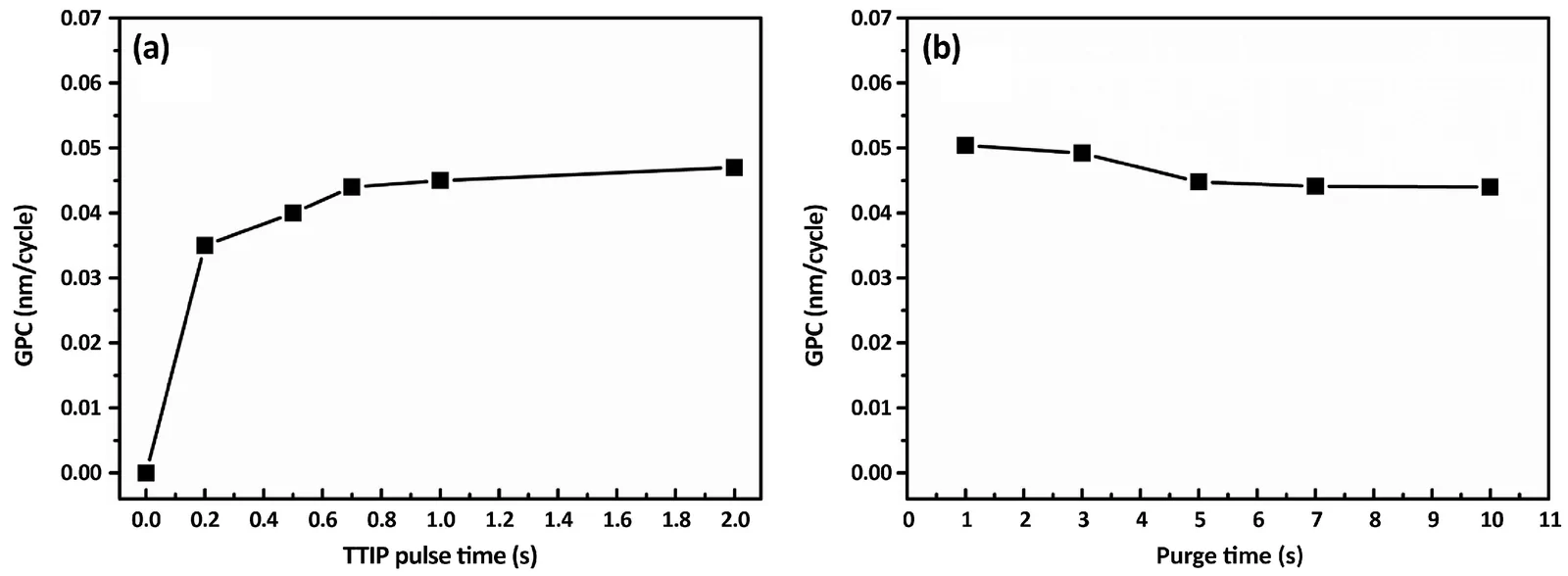
Plasma-enhanced atomic layer deposition (PE-ALD) saturation behavior analysis of TiO_2_ film growth. Saturation behavior as a function of (**a**) source pulse time and (**b**) purge time.

Figure 4 shows the growth per cycle (GPC) as a function of the TTIP pulse time and purge time to confirm the self-limiting growth behavior of the TiO_2_ films in the PE-ALD process.

Figure 4a shows the variation in GPC with TTIP pulse time. Film thickness was measured by spectroscopic ellipsometry, and GPC was calculated by dividing the measured thickness by the number of cycles. As the pulse time increases, GPC increases sharply and then reaches a stable saturation value of approximately 0.045 nm/cycle after 1.0 s. At short pulse times, the precursor dose is insufficient to fully cover the available reactive sites on the substrate surface, and GPC therefore increases with increasing pulse time. In contrast, for pulse times exceeding 1.0 s, the surface reactive sites become saturated, and no additional reaction occurs even if additional precursor is supplied, resulting in a constant GPC. This behavior is characteristic of ALD self-limiting surface chemistry and demonstrates the inherent ALD feature that deposition proceeds uniformly by approximately one layer per cycle [17].

Figure 4b shows the variation in GPC with TTIP purge time. With increasing purge time, GPC decreases slightly and converges to a saturated value of approximately 0.045 nm/cycle, reaching a fully saturated regime at purge times ≥ 5 s. For purge times shorter than 5 s, the unreacted precursor and reaction byproducts may not be completely removed from the chamber. These residual species can react with the oxygen source in the gas phase, resulting in partial chemical vapor deposition (CVD)-like growth in addition to the intrinsic surface-limited ALD reaction, which leads to a slightly higher measured GPC at purge times of <5 s. At sufficiently long purge times (≥5 s), the residual precursor and reaction byproducts are effectively removed, indicating that growth proceeds predominantly through the ALD mechanism without a CVD contribution [26]. Based on these results, a TTIP pulse time of 1 s and purge time of 5 s were selected as the optimal process conditions. The same precursor injection and purge times were applied in DP-ALD and RP-ALD; however, because remote plasma, by design, separates the plasma-generation region from the reaction chamber, a higher radio-frequency (RF) power than that used in direct plasma is required to deliver radicals to the substrate without quenching. Accordingly, the plasma conditions were optimized independently to ensure stable self-limiting growth for each method.

**Figure 5:**
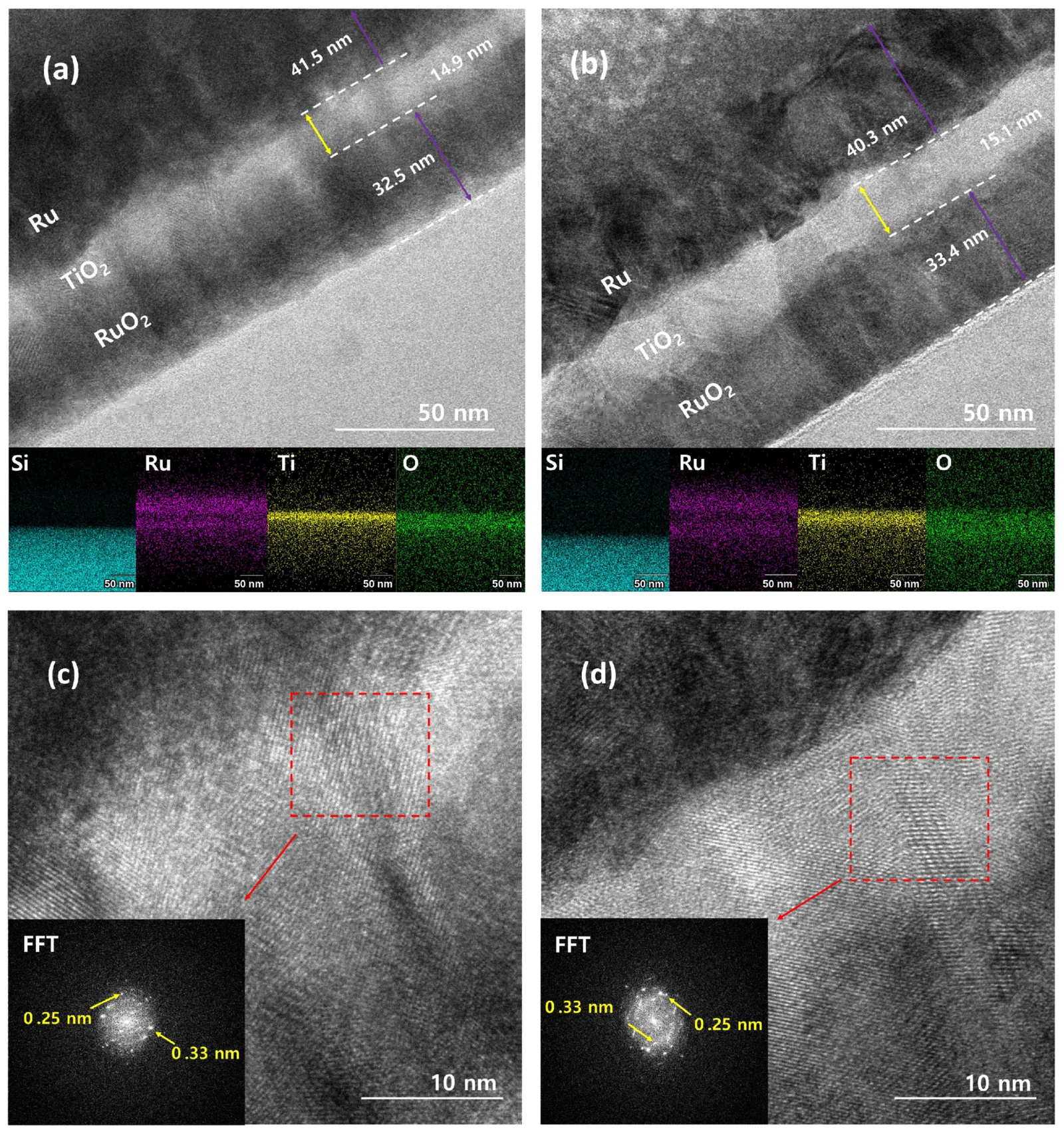
Cross-sectional transmission electron microscopy (TEM) images, energy-dispersive X-ray spectroscopy (EDS) elemental mapping, and high-resolution (HR-TEM)/fast Fourier transform (FFT) diffraction pattern results of the MIM capacitors fabricated by DP-ALD and RP-ALD. (**a**,**b**) Cross-sectional TEM images and EDS elemental maps of the DP-ALD- and RP-ALD-fabricated capacitors, respectively. (**c**,**d**) HR-TEM images and FFT diffraction patterns of the DP-ALD- and RP-ALD-fabricated capacitors, respectively.

Figure 5a and Figure 5b show the 50 nm-scale cross-sectional TEM images of the DP-ALD- and RP-ALD-fabricated capacitors, respectively, along with the corresponding EDS maps of Si K, Ru L, Ti K, and O K acquired from the same regions. In both samples, the Ru and Ti signals were spatially separated in the EDS maps, confirming a RuO_2_/TiO_2_/Ru stacked structure. In the DP-ALD-fabricated capacitor (Figure 5a), the RuO_2_/TiO_2_ interface was locally nonuniform, and the upper and lower interfaces of the TiO_2_ layer were therefore not clearly distinguished. By contrast, in the RP-ALD-fabricated capacitor (Figure 5b), the interface was comparatively uniform, and both the upper and lower TiO_2_ interfaces were clearly observed. This difference in interface morphology likely arises from the direct ion bombardment of the substrate during DP-ALD, which may affect the interface and the initial growth layer [30]. Figure 5c,d show the 10 nm-scale high-resolution transmission electron microscopy (HR-TEM) images of the TiO_2_ films deposited by DP-ALD and RP-ALD, respectively, along with the corresponding fast Fourier transform (FFT) diffraction patterns obtained from the TiO_2_ regions marked by red squares. In both samples, clear lattice fringes were observed throughout the TiO_2_ layer, with no evidence of an amorphous region. The FFT patterns exhibited distinct spots in both cases, indicating that the analyzed regions were crystalline. The lattice spacings measured from the FFT patterns were 0.33 and 0.25 nm, corresponding to the (110) plane (0.325 nm) and the (101) plane (0.249 nm) of rutile TiO_2_, respectively. Identical lattice spacings were observed for both the DP-ALD and RP-ALD-fabricated capacitors, likely because the underlying RuO_2_ shares the rutile structure with TiO_2_ and thus may provide an epitaxial template [41].

**Figure 6:**
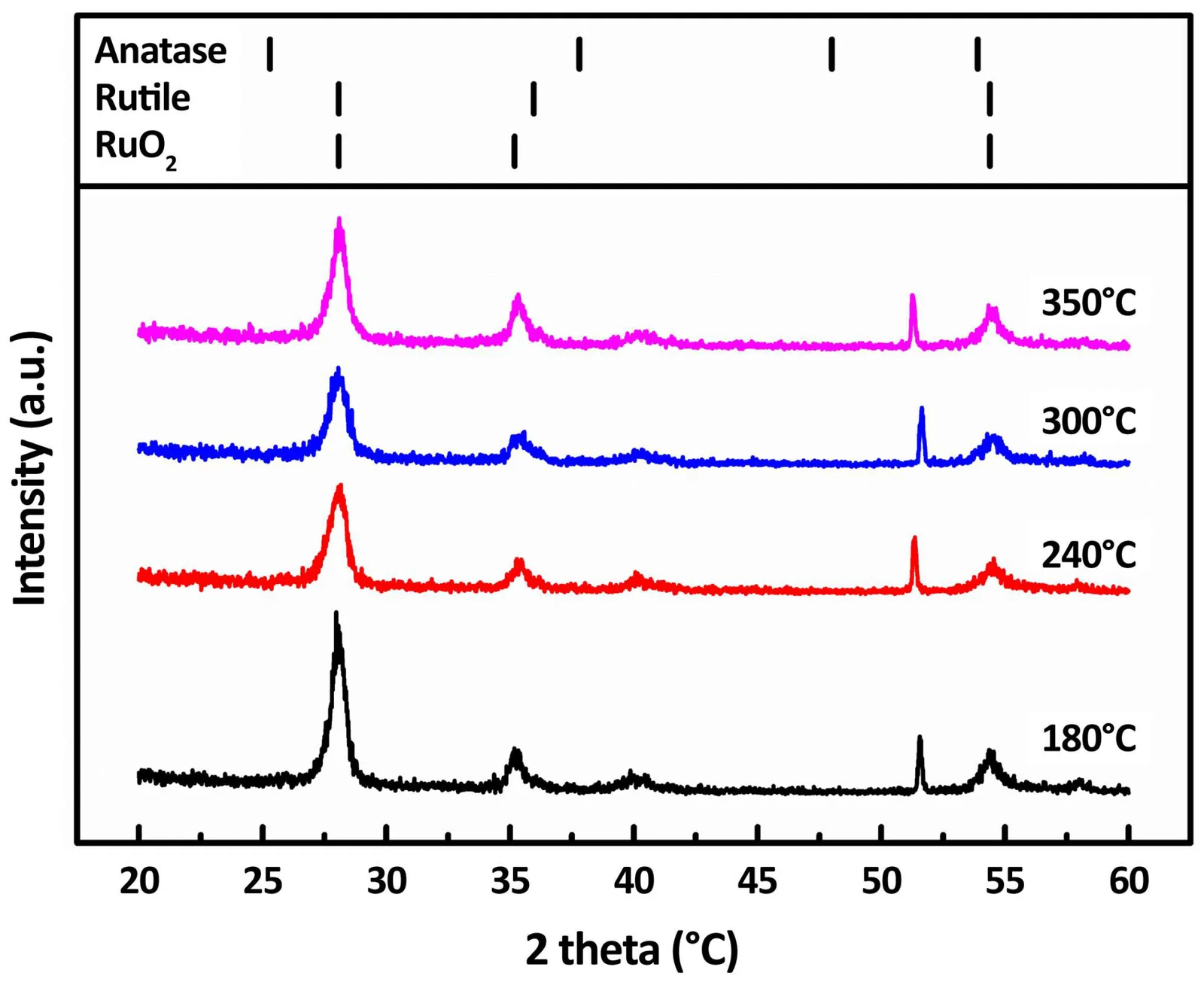
GIXRD results of TiO_2_ films as a function of deposition temperature (180, 240, 300, and 350 °C).

Figure 6 presents the GIXRD results for PE-ALD-deposited TiO_2_ films as a function of deposition temperature. Across the entire temperature range (180–350 °C), all samples exhibited diffraction peaks at similar positions near 28°, 35°, 40°, and 54–55°, consistent with the reference peak positions of rutile TiO_2_ and RuO_2_, indicated at the top of the graph. No distinct peaks were observed at the reference positions of anatase, confirming that, under the present experimental conditions, rutile TiO_2_ formed rather than the anatase phase [15,18]. Although TiO_2_ generally tends to form preferentially as anatase at relatively low temperatures [17], no anatase peaks were detected even at 180 °C. This result is likely attributable to the influence of the underlying RuO_2_ electrode on phase formation. Because RuO_2_ and rutile TiO_2_ share the same rutile structure and have similar lattice parameters, with a lattice mismatch of ~2%, this lattice matching suggests that the RuO_2_ layer acted as a template (seed layer) for the crystal structure of overlying TiO_2_ [15,21]. Thus, during nucleation, TiO_2_ likely grew in a rutile structure aligned with underlying RuO_2_, leading to predominantly rutile TiO_2_ over the studied deposition-temperature range. In addition, the highly reactive oxygen radicals supplied during PE-ALD may partially compensate for the activation energy required for surface reactions at low temperatures, thereby contributing to rutile-phase formation.

With increasing deposition temperature, the main peak near 28° (an overlapping RuO_2_/rutile TiO_2_ peak) remained relatively intense across the entire temperature range, and the additional peaks near 35° and 54–55° were also present at most temperatures. From 240 to 350 °C, the peak positions and shapes showed no discernible changes with the deposition temperature, indicating that the rutile structure of the underlying RuO_2_ electrode acted as a seed layer and enabled the stable formation of rutile TiO_2_ over the investigated deposition temperature range [42]. At 180 °C, however, the peak width appeared narrower than those at other temperatures. This narrowing may result from deposition below the lower limit of the ALD temperature window, where hindered self-limiting reactions may promote partial CVD-like growth and lead to abnormal film growth [43].

**Figure 7:**
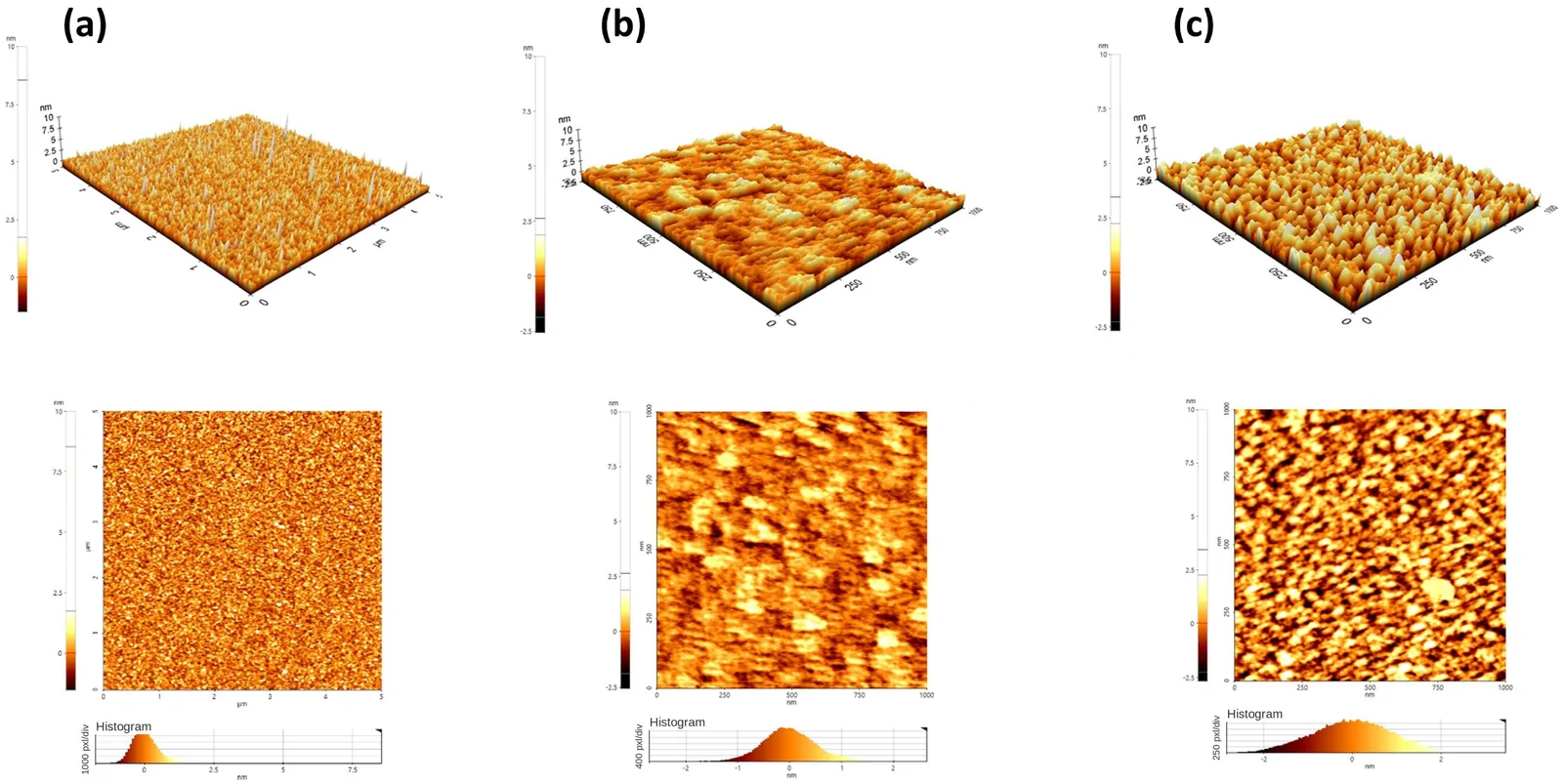
Atomic force microscopy (AFM) images of the RuO_2_/TiO_2_ surface morphology. (**a**) RuO_2_ bottom electrode (5 × 5 µm^2^). (**b**) DP-ALD TiO_2_ (1 × 1 µm^2^). (**c**) RP-ALD TiO_2_ (1 × 1 µm^2^).

Figure 7 compares the surface morphologies of the TiO_2_ films deposited by DP-ALD and RP-ALD on a RuO_2_ bottom electrode, determined through AFM. Because the RuO_2_ scan was intended to capture the macroscopic seed-layer morphology, whereas the TiO_2_ scans were intended to resolve the fine-scale grain structure, the two surfaces were examined at different length scales. For the RuO_2_ bottom electrode (Figure 7a), scanning was performed over a relatively large area (5 × 5 µm^2^) to capture the overall surface morphology. The TiO_2_ films deposited on RuO_2_ were then examined in tapping mode over a smaller area (1 × 1 µm^2^) to characterize the fine surface structure in greater detail. Accordingly, the roughness values obtained for RuO_2_ and TiO_2_ are not intended for direct quantitative comparison.

The RuO_2_ bottom electrode (Figure 7a) exhibited Ra = 0.365 nm and Rq = 0.498 nm over the 5 × 5 µm^2^ area, provided here as contextual background on the seed-layer morphology. For DP-ALD TiO_2_ (Figure 7b), relatively large grains with smooth, rounded surface profiles were nonuniformly distributed and formed blunt grain features that did not follow the underlying RuO_2_ surface morphology. In contrast, RP-ALD TiO_2_ (Figure 7c) showed fine, uniformly sized grains densely covering the surface, with growth that more closely followed the RuO_2_ morphology.

For DP-ALD TiO_2_, the surface roughness values were Ra = 0.400 nm and Rq = 0.511 nm, whereas those for RP-ALD TiO_2_ were Ra = 0.718 nm and Rq = 0.893 nm. The higher roughness of the RP-ALD film, exceeding even that of the RuO_2_ seed layer itself, may be related to the absence of pronounced ion-induced planarization during growth: because the growing TiO_2_ surface is not directly exposed to energetic ion bombardment, the roughness intrinsic to TiO_2_ grain nucleation appears to be superimposed on the existing grain-boundary topography of the RuO_2_ seed rather than being smoothed out, consistent with the finer-grained morphology observed in the 3D AFM topography (Figure 7c). In contrast, the DP-ALD film forms larger, rounded grains that are likely smoothed by ion bombardment during growth, resulting in a comparatively lower roughness despite the more energetic plasma conditions [30,44].

**Figure 8:**
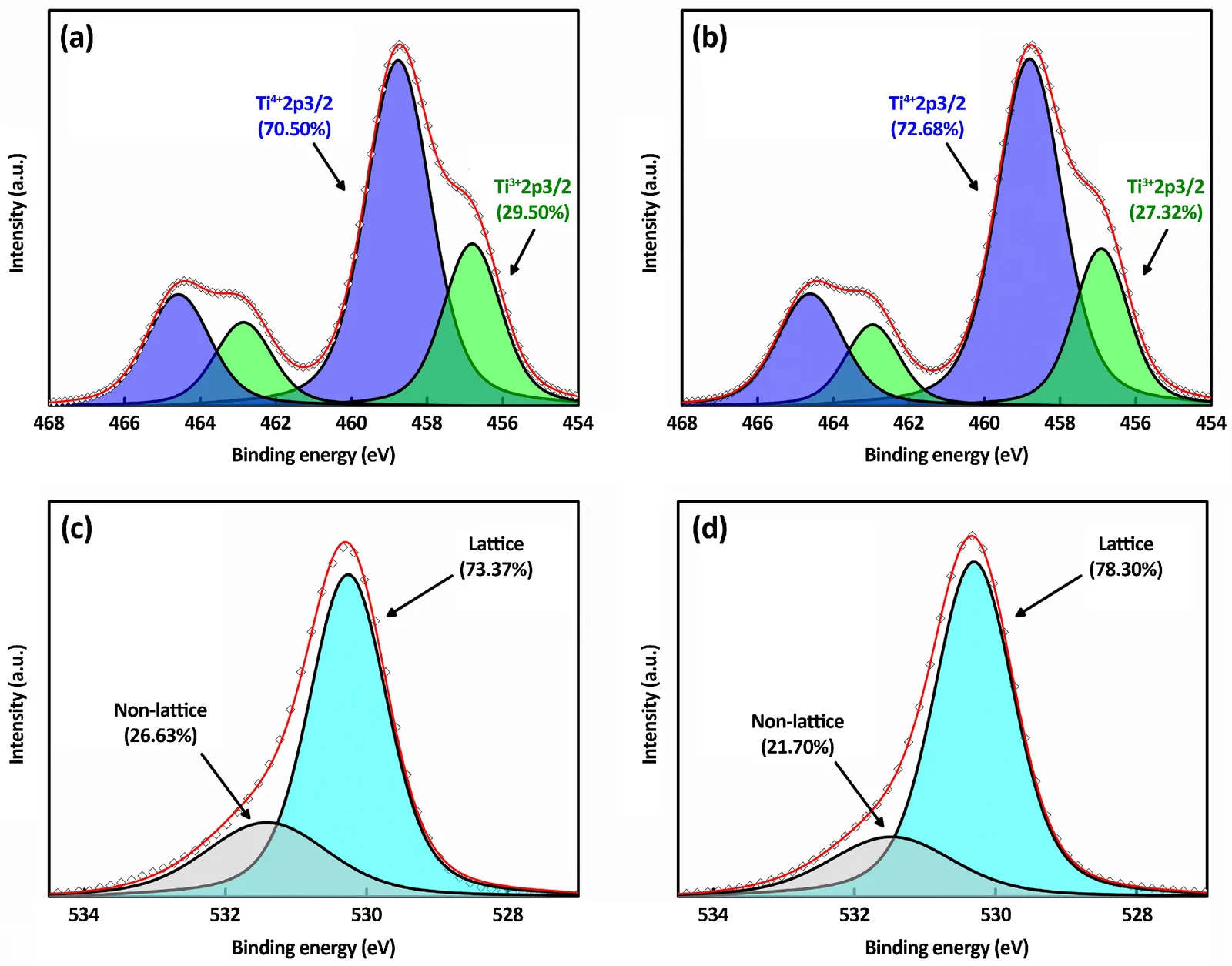
X-ray photoelectron (XP) spectra of the DP-ALD and RP-ALD TiO_2_ films: (**a**) DP-ALD Ti 2p, (**b**) RP-ALD Ti 2p, (**c**) DP-ALD O 1s, and (**d**) RP-ALD O 1s.

Figure 8 presents the XPS results of the chemical states of the TiO_2_ films deposited by DP-ALD and RP-ALD. Both specimens were measured after removing surface contamination under identical Ar^+^ ion etching conditions (500 eV, 40 s).

In the Ti 2p spectra, the Ti^4+^ 2p3/2 and Ti^3+^ 2p3/2 components were deconvoluted, and their relative ratios were quantified based on the 2p3/2 region. In the spectrum of the DP-ALD TiO_2_ film, Ti^4+^ 2p3/2 was observed at 459.0 eV and Ti^3+^ 2p3/2 at 456.8 eV, with relative ratios of 70.50% and 29.50%, respectively. In the spectrum of the RP-ALD TiO_2_ film, Ti^4+^ 2p3/2 was observed at 458.2 eV and Ti^3+^ 2p3/2 at 456.3 eV, with ratios of 72.68% and 27.32%, respectively, indicating a higher Ti^4+^ fraction than that observed in the DP-ALD film. Because Ti^3+^ is a reduced state associated with oxygen vacancies, the lower Ti^3+^ fraction in the RP-ALD film suggests a lower oxygen-vacancy concentration [45]. Although Ar^+^ bombardment may partially alter the measured Ti^3+^ fraction through preferential oxygen sputtering, this effect is expected to be comparable for both specimens given the identical etching conditions, and is therefore not expected to substantially affect the relative comparison between the two films.

The O 1s spectra were deconvoluted into lattice oxygen and nonlattice oxygen components. For the DP-ALD TiO_2_ film, the peak for lattice oxygen appeared at 530.3 eV and that for nonlattice oxygen at 531.4 eV, with relative ratios of 73.37% and 26.63%, respectively. For the RP-ALD TiO_2_ film, the peak for lattice oxygen appeared at 529.8 eV and that for nonlattice oxygen at 530.9 eV, with ratios of 78.30% and 21.70%, respectively, indicating a lower nonlattice oxygen fraction than that observed for the DP-ALD film. Because nonlattice oxygen is associated with oxygen vacancies and incomplete oxygen bonding, these results suggest that the RP-ALD film had a composition similar to stoichiometric TiO2 [45,46].

Together, the Ti 2p and O 1s results suggest that RP-ALD produces the TiO_2_ film with fewer oxygen vacancies than those observed in the DP-ALD film. This difference is likely related to the reduced defect formation under remote plasma conditions, where ion bombardment of the growth surface is mitigated in relation to those under direct plasma conditions [28,29,30,31].

### 3.2. MIM Device Electrical Characteristics

**Figure 9:**
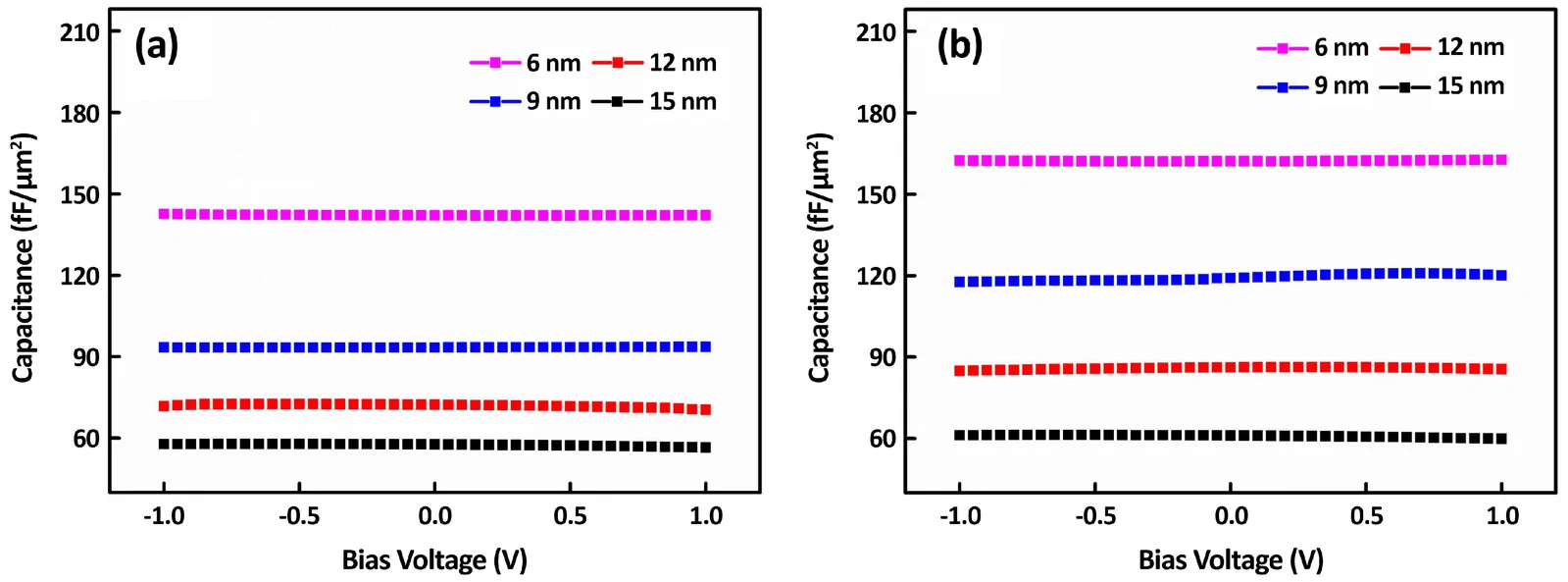
1 MHz C–V characteristics of the MIM capacitors as a function of TiO_2_ thickness (6, 9, 12, and 15 nm) deposited by (**a**) DP-ALD and (**b**) RP-ALD.

Figure 9 shows the capacitance–voltage (C–V) characteristics of the MIM capacitors as a function of TiO_2_ thickness for the films deposited by DP-ALD and RP-ALD. Measurements were performed at 1 MHz for TiO_2_ thicknesses of 6, 9, 12, and 15 nm, with a voltage sweep from −1 V to +1 V in 0.05 V steps. For both processes and all thicknesses, the C–V curves were nearly flat and parallel to the voltage axis, with minimal capacitance variation with applied voltage. This behavior indicates stable dielectric operation within the measurement range, consistent with reliable capacitor behavior without pronounced voltage-dependent effects such as charge injection.

For the DP-ALD-fabricated capacitor (Figure 9a), as the film thickness increased from 6 to 15 nm, the capacitance density decreased from approximately 142 to 57 fF/µm^2^. This behavior is consistent with the parallel-plate capacitor relationship, in which capacitance is inversely proportional to dielectric thickness: as the dielectric thickness increases, the electrode separation increases, reducing the capacitance per unit area. The RP-ALD-fabricated capacitor (Figure 9b) exhibited similarly flat C–V behavior, with capacitance inversely proportional to thickness; as the thickness increased from 6 to 15 nm, the capacitance density decreased from approximately 162 to 61 fF/µm^2^.

The C–V curves obtained for both processes exhibited a similar, nearly voltage-independent (flat) response; however, at the same thickness, the RP-ALD process consistently showed a higher capacitance density than the DP-ALD process. At 15 nm, the DP-ALD process yielded ~57 fF/µm^2^, whereas the RP-ALD process yielded ~61 fF/µm^2^, representing an approximate 7% increase for the RP-ALD process. Because capacitance density at a fixed thickness is proportional to the dielectric constant of the material, this difference suggests that the TiO_2_ films deposited by RP-ALD have a higher dielectric constant than those by DP-ALD [47].

**Figure 10:**
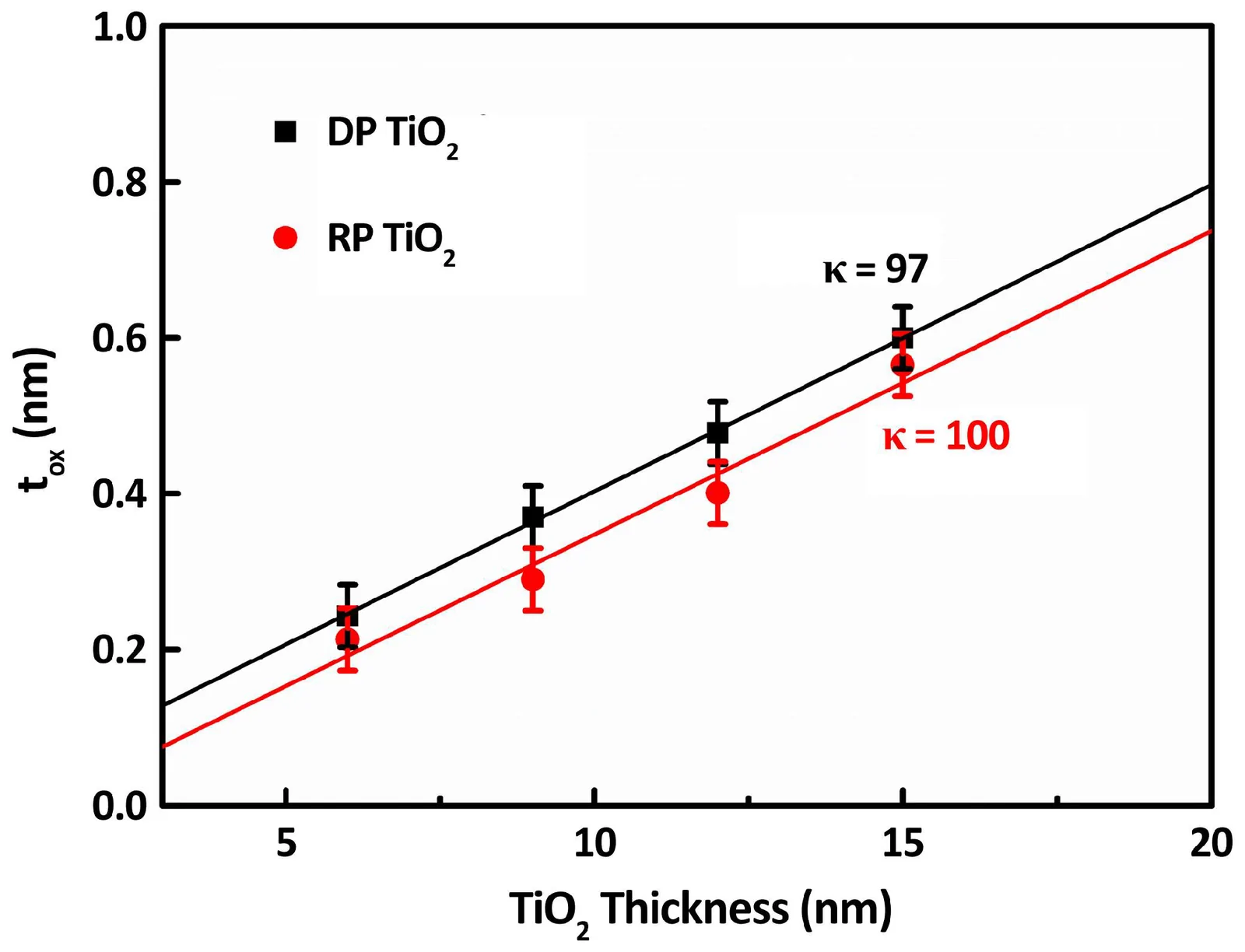
Equivalent oxide thickness (EOT) as a function of physical thickness for MIM capacitors fabricated by DP-ALD and RP-ALD.

Figure 10 presents the equivalent oxide thickness (EOT) analysis of the MIM capacitors as a function of the physical thickness of the TiO_2_ films deposited by DP-ALD and RP-ALD. The TiO_2_ dielectric constant (κ) was calculated from the measured capacitance using Equation (1).

(1) $$\kappa =\frac{C\cdot t_{TiO2}}{\varepsilon_{0}\cdot A}$$

where C is the measured capacitance, t_TiO2_ is the physical thickness of the TiO_2_ film, ε_0_ is the vacuum permittivity, and A is the electrode area. The EOT for each thickness was obtained by substituting the calculated dielectric constant into Equation (2).

(2) $$EOT=\frac{\varepsilon_{SiO2}}{\kappa }\cdot t_{TiO2}$$

where ε_SiO2_ is the dielectric constant of SiO_2_ (3.9). According to Equation (2), EOT varies linearly with TiO_2_ thickness, which can be expressed by the linear relation in Equation (3).

(3) $$EOT=a\cdot t_{TiO2}+b$$

Here, slope a corresponds to 3.9/κ; therefore, the intrinsic dielectric constant of TiO_2_ can be obtained from the slope of the EOT–thickness line using κ = 3.9/a. The y-intercept b represents the contribution of a low-dielectric-constant interfacial layer between the electrode and TiO_2_ [4].

For both processes, over the 6–15 nm thickness range, EOT followed a linear dependence on thickness, and both fits yielded y-intercepts close to 0, indicating that an interfacial layer, if present, contributes only minimally to the capacitance degradation. Linear fitting gave a slope of ~0.040 for the DP-ALD process, corresponding to a dielectric constant (κ) of approximately 97. For the RP-ALD process, the slope was ~0.039, giving a slightly higher κ of approximately 100. This result is consistent with the well-developed rutile structure observed by GIXRD and agrees with the results in Figure 9, where the RP-ALD process showed a modestly higher capacitance at the same thickness.

**Figure 11:**
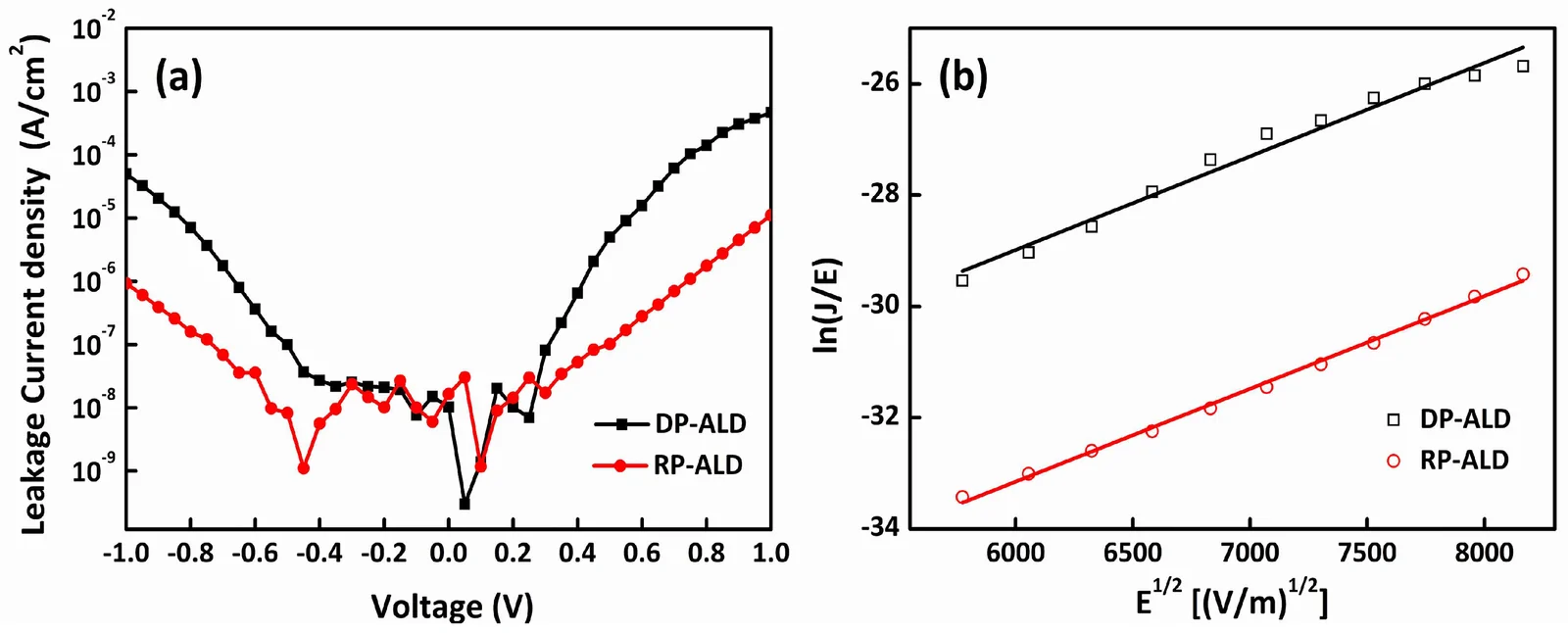
(**a**) Leakage current density–voltage (J–V) characteristics and (**b**) the corresponding Poole-Frenkel plot [ln(J/E) vs. E^1/2^] of the 15 nm TiO_2_ MIM capacitors fabricated by DP-ALD and RP-ALD.

Figure 11 shows (a) the leakage current density–voltage (J–V) characteristics and (b) the corresponding Poole-Frenkel (PF) plot of the 15 nm TiO_2_ MIM capacitors fabricated by DP-ALD and RP-ALD. Because TiO_2_ has a narrow bandgap (~3.0 eV), tunneling-dominated leakage increases sharply as the film becomes thinner; consequently, differences in leakage associated with process-induced defect densities may be partially masked in thinner films [48]. To more clearly compare leakage behavior of DP-ALD and RP-ALD films, the J–V characteristics in this section were therefore evaluated using the 15 nm TiO_2_ device, for which the tunneling contribution is smallest among the studied thicknesses. Measurements were performed by sweeping the voltage from −1 V to +1 V in 0.05 V steps. Even at the same thickness, the two processes produced distinct leakage levels: across the entire voltage range, the DP-ALD-fabricated capacitor exhibited a higher leakage current density than the RP-ALD-fabricated capacitor. To quantitatively examine the origin of this difference, the J–V data over the 0.5 to 1.0 V range were fitted to the Poole-Frenkel model, which describes conduction governed by field-assisted emission of trapped electrons (Figure 11b). The Poole-Frenkel conduction is expressed in Equation (4) as

(4) $$J\propto E\;exp[\frac{-q(\varphi_{B}-\sqrt{\frac{qE}{\pi \varepsilon }})}{kT}]$$

where E is the electric field, φ_B_ is the trap potential barrier height (trap depth), ε is the dielectric permittivity of the film, and the remaining symbols are conventional physical constants. Both films showed a high linear correlation (R^2^ = 0.979 for DP-ALD, R^2^ = 0.997 for RP-ALD), consistent with trap-assisted emission as the operative conduction mechanism in both films. The two fitted lines showed closely comparable slopes, suggesting that DP-ALD and RP-ALD are governed by the same conduction mechanism, while the DP-ALD line was offset to substantially higher current density at every field value, consistent with a higher trap density in the DP-ALD film [49]. The observed behavior is consistent with differences in film quality associated with the plasma-generation approach. In the DP-ALD process, plasma is ignited directly in the deposition chamber, allowing the growing film surface to be directly exposed to high-energy ion bombardment. Such bombardment may contribute to increased leakage pathways through surface damage and oxygen-vacancy formation. Conversely, in the RP-ALD process, plasma is generated outside the chamber and mainly reactive radicals are delivered to the growth region; thus, both the ion flux and ion energy at the growth surface are expected to be lower [28,29]. As a result, the RP-ALD-fabricated capacitor is inferred to have a lower defect density, suppressing leakage across the entire voltage range. At 0.8 V for the 15 nm device, the DP-ALD-fabricated capacitor exhibited a leakage current density of approximately 1.41 × 10^−4^ A/cm^2^, whereas the RP-ALD-fabricated capacitor showed a much lower value of approximately 1.76 × 10^−6^ A/cm^2^.

**Figure 12:**
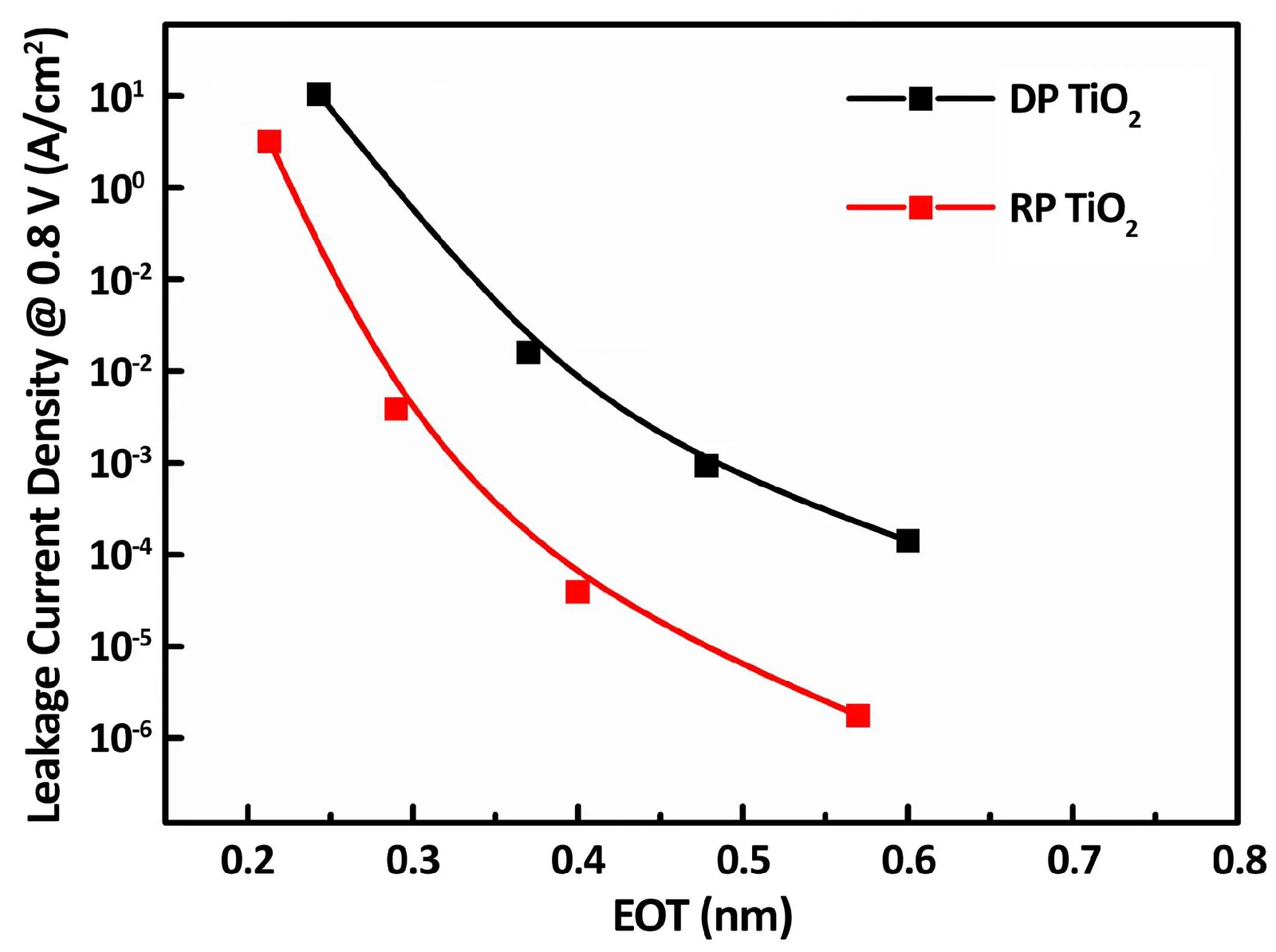
J–EOT characteristics of the TiO_2_ MIM capacitors fabricated by DP-ALD and RP-ALD. The leakage current density was measured at +0.8 V for each EOT value.

Figure 12 shows the J–EOT characteristics of the TiO_2_ MIM capacitors fabricated by DP-ALD and RP-ALD, with the leakage current density measured at +0.8 V plotted as a function of EOT for each plasma type.

For both processes, the leakage current density decreased with increasing EOT. For the DP-ALD process, as EOT increased from 0.24 to 0.60 nm, the leakage current density decreased from approximately 10 A/cm^2^ to 1.4 × 10^−4^ A/cm^2^. For the RP-ALD process, as EOT increased from 0.21 to 0.57 nm, the leakage current density decreased from approximately 3 A/cm^2^ to 1.76 × 10^−6^ A/cm^2^. Notably, the RP-ALD-fabricated capacitor showed a leakage current density approximately two orders of magnitude lower than that of the DP-ALD-fabricated capacitor despite its lower EOT. This improvement is likely related to suppressed trap-defect formation because the growing film in the RP-ALD process is not directly exposed to plasma, thereby improving the trade-off between EOT and leakage current while maintaining the high dielectric constant of the rutile TiO_2_ [50].

Table 1 compares the electrical properties of the RP-ALD-fabricated capacitor obtained in this study with those of previously reported MIM capacitors employing TiO_2_ dielectrics. The reported dielectric constants ranged from 79 to 130, while the leakage current densities ranged from 1.00 × 10^−3^ to 7.50 × 10^1^ A/cm^2^. The RP-ALD-fabricated capacitor investigated in this study exhibited a leakage current density of 1.76 × 10^−6^ A/cm^2^ and a dielectric constant of 100. The leakage current density is more than one order of magnitude lower than previously reported values for capacitors with similar dielectric constants [51,52,53,54,55]. Therefore, as shown in Figure 12, maintaining a low leakage current at a reduced EOT highlights the advantages of RP-ALD for achieving the low leakage and high capacitance characteristics required for high-density DRAM capacitors.

## 4. Conclusions

In this study, RuO_2_/TiO_2_/Ru MIM capacitors were fabricated by depositing TiO_2_ dielectric films on a RuO_2_ bottom electrode using DP-ALD and RP-ALD, and the effects of these two plasma excitation modes on the structural, chemical, and electrical properties of the films were systematically compared.

The RuO_2_ bottom electrode deposited by reactive DC sputtering exhibited improved crystallinity with increasing substrate temperature and Ar/O_2_ flow ratio, and it formed a well-defined crystalline rutile structure with a (110) preferred orientation at 300 °C and an Ar:O_2_ ratio of 7:3. Because this RuO_2_ electrode has lattice parameters similar to those of rutile TiO_2_, it likely acted as a seed layer for rutile TiO_2_ growth. Accordingly, GIXRD and HR-TEM/FFT analyses confirmed the formation of phase-pure rutile TiO_2_ over a wide deposition-temperature range of 180–350 °C.

Comparison of the TiO_2_ films deposited by DP-ALD and RP-ALD via XPS showed that the RP-ALD film exhibited a lower Ti^3+^ and nonlattice oxygen fraction (21.70%) than the DP-ALD film. These results suggest a lower concentration of oxygen vacancies and improved stoichiometric stability. Consistent with these findings, TEM interface analysis showed a relatively uniform RuO_2_/TiO_2_ interface in the RP-ALD film, with lattice fringes that remained continuous across the interface, suggesting that the growth-surface damage during deposition was less severe than that in DP-ALD.

Electrical characterization showed that the RP-ALD-fabricated MIM capacitor exhibited a dielectric constant of ~100 and a leakage current density of ~1.76 × 10^−6^ A/cm^2^ at 0.8 V. Compared with the DP-ALD-fabricated capacitor (dielectric constant of ~97; leakage current density of ~1.41 × 10^−4^ A/cm^2^), the RP-ALD-fabricated capacitor exhibited a slightly higher dielectric constant and a leakage current density approximately two orders of magnitude lower than the DP-ALD-fabricated capacitor. In the J–EOT analysis, the RP-ALD-fabricated capacitor also exhibited a lower leakage current density at a smaller EOT, confirming an improved EOT–leakage-current trade-off.

Together, these results suggest that the remote plasma method can suppress the generation of ion-induced defects during TiO_2_ deposition, thereby enabling low leakage current while preserving the high dielectric constant of rutile TiO_2_.

## Figures and Tables

**Table 1:** Electrical properties of TiO_2_-based MIM capacitors employing different electrode materials.

Bottom Electrode	Top Electrode	k-Value	Leakage Current [A/cm^2^]	Ref.
RuO_2_	Ru	100	1.76 × 10^−6^	This work
Pt	Pt	85.2	3 × 10^−5^	[51]
Ru/RuO_2_	TiN	79	1.00 × 10^−3^	[52]
MoO_2_ (MO)	RuO_2_	110.63	3.39 × 10^−4^	[53]
MoO_2_	RuO_2_	107	2.00 × 10^−3^	[54]
Ru	Ru	130	7.50 × 10^1^	[55]

## Data Availability

The data presented in this study are available within the article.

## Abbreviations

The following abbreviations are used in this manuscript:

DRAM	Dynamic random-access memory
ALD	Atomic layer deposition
MIM	Metal–insulator–metal
RP-ALD	Remote plasma ALD
DP-ALD	Direct plasma ALD
EOT	Equivalent oxide thickness
ZAZ	ZrO_2_/Al_2_O_3_/ZrO_2_
PE-ALD	Plasma-enhanced atomic layer deposition

## References

[1] Kim S.K., Popovici M. (2018). Future of dynamic random-access memory as main memory. MRS Bull..

[2] Jeon W. (2020). Recent advances in the understanding of high-k dielectric materials deposited by atomic layer deposition for dynamic random-access memory capacitor applications. J. Mater. Res..

[3] Hwang C.S. (2015). Prospective of semiconductor memory devices: From memory system to materials. Adv. Electron. Mater..

[4] Kim S.K., Lee S.W., Han J.H., Lee B., Han S., Hwang C.S. (2010). Capacitors with an equivalent oxide thickness of <0.5 nm for nanoscale electronic semiconductor memory. Adv. Funct. Mater..

[5] Kim S.E., Sung J.Y., Jeon J.D., Jang S.Y., Lee H.M., Moon S.M., Kang J.G., Lim H.J., Jung H.-S., Lee S.W. (2023). Toward advanced high-k and electrode thin films for DRAM capacitors via atomic layer deposition. Adv. Mater. Technol..

[6] Spessot A., Oh H. (2020). 1T-1C dynamic random access memory status, challenges, and prospects. IEEE Trans. Electron Devices.

[7] Choi J.H., Mao Y., Chang J.P. (2011). Development of hafnium based high-k materials—A review. Mater. Sci. Eng. R Rep..

[8] Robertson J., Wallace R.M. (2015). High-k materials and metal gates for CMOS applications. Mater. Sci. Eng. R Rep..

[9] Cha S.H., An C.H., Cho S.T., Kim D.-G., Kwon D.S., Lim J.I., Jeon W., Hwang C.S. (2019). Scaling the equivalent oxide thickness by employing a TiO_2_ thin film on a ZrO_2_–Al_2_O_3_-based dielectric for further scaling of dynamic random access memory. Phys. Status Solidi RRL.

[10] An C.H., Lee W., Kim S.H., Cho C.J., Kim D.-G., Kwon D.S., Cho S.T., Cha S.H., Lim J.I., Jeon W. (2019). Controlling the electrical characteristics of ZrO_2_/Al_2_O_3_/ZrO_2_ capacitors by adopting a Ru top electrode grown via atomic layer deposition. Phys. Status Solidi RRL.

[11] Wilk G.D., Wallace R.M., Anthony J.M. (2001). High-κ gate dielectrics: Current status and materials properties considerations. J. Appl. Phys..

[12] Kim S.K., Choi G.-J., Lee S.Y., Seo M., Lee S.W., Han J.H., Ahn H.-S., Han S., Hwang C.S. (2008). Al-doped TiO_2_ films with ultralow leakage currents for next generation DRAM capacitors. Adv. Mater..

[13] Lee S., Han G., Kim K.H., Shim D., Go D., An J. (2024). High-performance TiO_2_/ZrO_2_/TiO_2_ thin film capacitor by plasma-assisted atomic layer annealing. ACS Appl. Mater. Interfaces.

[14] Hudec B., Hušeková K., Tarre A., Han J.H., Han S., Rosová A., Lee W., Kasikov A., Song S.J., Aarik J. (2011). Electrical properties of TiO_2_-based MIM capacitors deposited by TiCl_4_ and TTIP based atomic layer deposition processes. Microelectron. Eng..

[15] Fröhlich K., Aarik J., Ťapajna M., Rosová A., Aidla A., Dobročka E., Hušková K. (2009). Epitaxial growth of high-κ TiO_2_ rutile films on RuO_2_ electrodes. J. Vac. Sci. Technol. B.

[16] Tang H., Prasad K., Sanjinès R., Schmid P.E., Lévy F. (1994). Electrical and optical properties of TiO_2_ anatase thin films. J. Appl. Phys..

[17] Ritala M., Leskelä M., Niinistö L., Haussalo P. (1993). Titanium isopropoxide as a precursor in atomic layer epitaxy of titanium dioxide thin films. Chem. Mater..

[18] Jeon J., Kim T., Jang M., Chung H.K., Kim S.-C., Won S.O., Park Y., Choi B.J., Chung Y.J., Kim S.K. (2024). High-temperature atomic layer deposition of rutile TiO_2_ films on RuO_2_ substrates: Interfacial reactions and dielectric performance. Chem. Mater..

[19] Jeon J., Kim J., Ye S., Kim S.K. (2026). Rutile without substrate limitations: Top-interface-driven crystallization of TiO_2_. Adv. Mater..

[20] Jeon J., Ye S., Kim J., Kim S.K. (2026). Beyond ZrO_2_: Rutile TiO_2_ as the dielectric platform for next-generation DRAM capacitors. ACS Appl. Electron. Mater..

[21] Kim S.K., Kim W.D., Kim K.M., Hwang C.S., Jeong J. (2004). High dielectric constant TiO_2_ thin films on a Ru electrode grown at 250 °C by atomic-layer deposition. Appl. Phys. Lett..

[22] Kwon D.S., Jeon W., Kim D.G., Kim T.K., Seo H., Lim J., Hwang C.S. (2021). Improved properties of the atomic layer deposited Ru electrode for dynamic random-access memory capacitor using discrete feeding method. ACS Appl. Mater. Interfaces.

[23] Han J.H., Han S., Lee W., Lee S.W., Kim S.K., Gatineau J., Dussarrat C., Hwang C.S. (2011). Improvement in the leakage current characteristic of metal-insulator-metal capacitor by adopting RuO_2_ film as bottom electrode. Appl. Phys. Lett..

[24] Nalawade S., Vondee E., Liu M., Chris-Okoro I., Cherono S., Kumar D., Aravamudhan S. (2025). Role of oxygen concentration in reactive sputtering of RuO_2_ thin films: Tuning surface chemistry for enhanced electrocatalytic performance. Crystals.

[25] Aarik J., Arroval T., Aarik L., Rammula R., Kasikov A., Mändar H., Hudec B., Hušeková K., Fröhlich K. (2013). Atomic layer deposition of rutile-phase TiO_2_ on RuO_2_ from TiCl_4_ and O_3_: Growth of high-permittivity dielectrics with low leakage current. J. Cryst. Growth.

[26] George S.M. (2010). Atomic layer deposition: An overview. Chem. Rev..

[27] Johnson R.W., Hultqvist A., Bent S.F. (2014). A brief review of atomic layer deposition: From fundamentals to applications. Mater. Today.

[28] Profijt H.B., Potts S.E., van de Sanden M.C.M., Kessels W.M.M. (2011). Plasma-assisted atomic layer deposition: Basics, opportunities, and challenges. J. Vac. Sci. Technol. A.

[29] Knoops H.C.M., Faraz T., Arts K., Kessels W.M.M. (2019). Status and prospects of plasma-assisted atomic layer deposition. J. Vac. Sci. Technol. A.

[30] Arts K., Thepass H., Verheijen M.A., Puurunen R.L., Kessels W.M.M., Knoops H.C.M. (2021). Impact of ions on film conformality and crystallinity during plasma-assisted atomic layer deposition of TiO_2_. Chem. Mater..

[31] Kim K., Oh I.-K., Kim H., Lee Z. (2017). Atomic-scale characterization of plasma-induced damage in plasma-enhanced atomic layer deposition. Appl. Surf. Sci..

[32] Lo Nigro R., Schilirò E., Mannino G., Di Franco S., Roccaforte F. (2020). Comparison between thermal and plasma enhanced atomic layer deposition processes for the growth of HfO_2_ dielectric layers. J. Cryst. Growth.

[33] Heil S.B.S., van Hemmen J.L., Hodson C.J., Singh N., Klootwijk J.H., Roozeboom F., van de Sanden M.C.M., Kessels W.M.M. (2007). Deposition of TiN and HfO_2_ in a commercial 200 mm remote plasma atomic layer deposition reactor. J. Vac. Sci. Technol. A.

[34] Das C., Henkel K., Tallarida M., Schmeißer D., Gargouri H., Kärkkänen I., Schneidewind J., Gruska B., Arens M. (2015). Thermal and plasma enhanced atomic layer deposition of TiO_2_: Comparison of spectroscopic and electric properties. J. Vac. Sci. Technol. A.

[35] Yoo J.-H., Park W.-J., Kim S.-W., Lee G.-R., Kim J.-H., Lee J.-H., Uhm S.-H., Lee H.-C. (2023). Preparation of remote plasma atomic layer-deposited HfO_2_ thin films with high charge trapping densities and their application in nonvolatile memory devices. Nanomaterials.

[36] Kim J., Hwang I., Kim B., Lee W., Song J., Jung Y., Yoon C. (2025). Deposition of HfO_2_ by remote plasma ALD for high-aspect-ratio trench capacitors in DRAM. Nanomaterials.

[37] Tallarida M., Friedrich D., Städter M., Michling M., Schmeisser D. (2011). Growth of TiO_2_ with thermal and plasma enhanced atomic layer deposition. J. Nanosci. Nanotechnol..

[38] Hwang I., Kim J., Lee J., Jung Y., Yoon C. (2025). Memory devices with HfO_2_ charge-trapping and TiO_2_ channel layers: Fabrication via remote and direct plasma atomic layer deposition and comparative performance evaluation. Materials.

[39] Kim B., Kang T., Lee G., Jeon H. (2022). The effect of an annealing process on atomic layer deposited TiO_2_ thin films. Nanotechnology.

[40] Thornton J.A. (1974). Influence of apparatus geometry and deposition conditions on the structure and topography of thick sputtered coatings. J. Vac. Sci. Technol..

[41] Kim T., Jeon J., Ryu S.H., Chung H.K., Jang M., Lee S., Chung Y.J., Kim S.K. (2024). Atomic layer growth of rutile TiO_2_ films with ultrahigh dielectric constants via crystal orientation engineering. ACS Appl. Mater. Interfaces.

[42] Jeon J., Jang M., Ye S., Kim T., Kim S.-C., Won S.O., Kim S.K. (2025). Metastable rutile TiO_2_ growth on non-lattice-matched substrates via a sacrificial layer strategy. Small.

[43] Wei D., Hossain T., Garces N.Y., Nepal N., Meyer H.M., Kirkham M.J., Eddy C.R., Edgar J.H. (2013). Influence of atomic layer deposition temperatures on TiO_2_/n-Si MOS capacitor. ECS J. Solid State Sci. Technol..

[44] Xiong L., Hu J., Yang Z., Li X., Zhang H., Zhang G. (2022). Dielectric properties investigation of metal–insulator–metal (MIM) capacitors. Molecules.

[45] Bharti B., Kumar S., Lee H.-N., Kumar R. (2016). Formation of oxygen vacancies and Ti^3+^ state in TiO_2_ thin film and enhanced optical properties by air plasma treatment. Sci. Rep..

[46] Diebold U. (2003). The surface science of titanium dioxide. Surf. Sci. Rep..

[47] Zheng G., He Y.-L., Zhu B., Wu X., Zhang D.W., Ding S.-J. (2023). Improvement of voltage linearity and leakage current of MIM capacitors with atomic layer deposited Ti-doped ZrO_2_ insulators. IEEE Trans. Electron Devices.

[48] Robertson J. (2000). Band offsets of wide-band-gap oxides and implications for future electronic devices. J. Vac. Sci. Technol. B.

[49] Fröhlich K., Hudec B., Aarik J., Tarre A., Machajdík D., Kasikov A., Hušeková K., Gaži Š. (2011). Post-deposition processing and oxygen content of TiO_2_-based capacitors. Microelectron. Eng..

[50] Das D., Buyantogtokh B., Gaddam V., Jeon S. (2022). Sub 5 Å-EOT Hf_x_Zr_1−x_O_2_ for next-generation DRAM capacitors using morphotropic phase boundary and high-pressure (200 atm) annealing with rapid cooling process. IEEE Trans. Electron Devices.

[51] Eun S.M., Hwang J.H., Choi B.J. (2024). Reduction of leakage current and enhancement of dielectric properties of rutile-TiO_2_ film deposited by plasma-enhanced atomic layer deposition. Korean J. Mater. Res..

[52] Popovici M., Kim M.-S., Tomida K., Swerts J., Tielens H., Moussa A., Richard O., Bender H., Franquet A., Conard T. (2011). Improved EOT and leakage current for metal–insulator–metal capacitor stacks with rutile TiO_2_. Microelectron. Eng..

[53] Yoo S.M., Lee S., Hwang C., Jeon W. (2026). Improved molybdenum dioxide atomic layer deposition process by introducing pre-reduction agent. Adv. Electron. Mater..

[54] Hwang C., Kim Y.W., Park J., Kim M.H., Kim J.-S., Jeon W. (2024). In-situ crystallization of rutile-phased TiO_2_ via the template effect using MoO_2_ electrode for the metal-insulator-metal capacitor applications. J. Alloys Compd..

[55] Jithin M.A., Kolla L.G., Bhat N., Mohan S., Morozumi Y., Kaushal S. (2013). Pulsed DC magnetron sputtered rutile TiO_2_ films for next generation DRAM capacitors. MRS Online Proc. Libr..

